# Paroxysmal Nocturnal Hemoglobinuria Clones in Children with Acquired Aplastic Anemia: A Multicentre Study

**DOI:** 10.1371/journal.pone.0101948

**Published:** 2014-07-09

**Authors:** Fabio Timeus, Nicoletta Crescenzio, Daniela Longoni, Alessandra Doria, Luiselda Foglia, Sara Pagliano, Stefano Vallero, Valentina Decimi, Johanna Svahn, Giuseppe Palumbo, Antonio Ruggiero, Baldassarre Martire, Marta Pillon, Nicoletta Marra, Carlo Dufour, Ugo Ramenghi, Paola Saracco

**Affiliations:** 1 Pediatric Onco-Hematology, Regina Margherita Children’s Hospital, Turin, Italy; 2 Pediatric Hematology, University of Turin, Turin, Italy; 3 Pediatric Department MBBM Foundation S. Gerardo Hospital, Monza, Italy; 4 Hematology Unit, G. Gaslini Children’s Hospital, Genoa, Italy; 5 Pediatric Onco-Hematology Department, Bambin Gesù Children’s Hospital, Rome, Italy; 6 Pediatric Oncology, Policlinico Gemelli, Rome, Italy; 7 Department of Pediatrics, University of Bari, Bari, Italy; 8 Pediatric Onco-Hematology Unit, University Hospital of Padua, Padua, Italy; 9 Department of Pediatric Haemato-Oncology, Santobono-Pausilipon Hospital, Naples, Italy; University of Louisville, United States of America

## Abstract

A multicentre study evaluating the presence of glycosil phosphatidyl-inositol (GPI)-negative populations was performed in 85 children with acquired aplastic anemia (AA). A GPI-negative population was observed in 41% of patients at diagnosis, 48% during immune-suppressive therapy (IST), and 45% in patients off-therapy. No association was found between the presence of a GPI-negative population at diagnosis and the response to IST. In addition, the response rate to IST did not differ between the patients who were GPI-positive at diagnosis and later developed GPI-negative populations and the 11 patients who remained GPI-positive. Two patients with a GPI-negative population >10%, and laboratory signs of hemolysis without hemoglobinuria were considered affected by paroxysmal nocturnal hemoglobinuria (PNH) secondary to AA; no thrombotic event was reported. Excluding the 2 patients with a GPI-negative population greater than 10%, we did not observe a significant correlation between LDH levels and GPI-negative population size. In this study monitoring for laboratory signs of hemolysis was sufficient to diagnose PNH in AA patients. The presence of minor GPI-negative populations at diagnosis in our series did not influence the therapeutic response. As occasionally the appearance of a GPI-negative population was observed at cyclosporine (CSA) tapering or AA relapse, a possible role of GPI-negative population monitoring during IST modulation may need further investigation.

## Introduction

Paroxysmal nocturnal hemoglobinuria (PNH) is an acquired hematopoietic stem cell (HSC) disorder characterized by the clonal expansion of a *PIG-A* mutated stem cell and consequent defective synthesis of glycosil phosphatidyl-inositol-anchored proteins, complement-mediated hemolysis, increased incidence of thrombosis, and bone marrow failure. PNH and acquired aplastic anemia (AA) are closely related. Flow cytometry analysis (FCA) to diagnose PNH was proposed in 1996 by Hall and Rosse [1]. “Routine” CD55 and CD59-based assays are not sensitive below a clone size less than 1%, whereas two-color FCA [2] or fluorescent-labeled aerolysin (FLAER)-based assays [3], [4] are more sensitive. High resolution FCA has revealed the presence of very low numbers of GPI-negative granulocytes in normal subjects at an average frequency of 22/10^6^ [5]. A study on PIG-A mutations in CD34+ cells from PNH patients and normal controls showed clonal mutations in PNH patients and a polyclonal pattern in normal subjects, suggesting that in normal subjects PIG-A mutations occur in differentiated progenitors [6]. A relative resistance of the PNH HSC to the immune-mediated damage may explain the clonal expansion of PNH cells in AA [7] in a multi-step model together with other mechanisms, such as the deregulation of the *HMGA2* gene, conferring increased resistence to apoptosis. [8]. FCA has revealed a high incidence of minor GPI-negative populations in adult AA patients at diagnosis, predictive of a favourable response to immunosuppressive therapy (IST) [2], [9], [10]. Hemolytic PNH with frank hemoglobinuria, is a very rare disease in adults and extremely rare in children.

Only a few studies have so far evaluated GPI-negative populations in pediatric AA patients. Yoshida *et al* [11] demonstrated the presence of GPI-negative cells in 21.4% (population size 0.04–0.81%) of 103 children with AA. Scheinberg *et al* [12] in a series of 152 adults and 45 children with AA observed the presence of GPI-negative populations in 40% of patients. Timeus *et al* [13] demonstrated the presence of minor GPI-negative populations in 53% (population size 0.2–2.2%) of 17 AA patients studied at diagnosis. Sutton *et al* [14] observed a GPI-negative population in 9/23 severe AA and in 1/3 moderate AA. No correlation was found between GPI-negative populations and response to IST.

## Materials and Methods

The present multicentre longitudinal study started in 2008 and was performed on 85 children with AA diagnosed in 8 AIEOP (Italian Association of Pediatric Hematology-Oncology) Centres (age at diagnosis 1–17 years, median = 10.7, 46 severe AA, 30 very severe AA, 9 non severe AA, see Table 1). Written informed consent to perform diagnostic and follow up examinations required in the treatment protocol of acquired aplastic anemia (approved by the single center Ethical Committees) was obtained by patient’s parents at time of diagnosis. A specific informed consent form for this study was not required by Ethical Committees as the PNH analysis was a diagnostic test performed on peripheral blood during routine follow up of patients and was not a genetic test. This statement was confirmed by Comitato Etico Interaziendale OIRM-S.ANNA-ORDINE MAURIZIANO (prot n. 24316/C28.1; website: www.cittadellasalute.to.it).

**Table 1:** AIEOP centres participating to the study.

AIEOP CENTRES	N. PATIENTS AT DIAGNOSIS	N. PATIENTS IN IST	N. PATIENTS OFF THERAPY
**TURIN**	20	5*	4
**MONZA**	8	6	9
**GENOA**	5	11	4
**NAPLES**	3	1	1
**ROME BAMBIN GESU’ CHILDREN HOSPITAL**	2	0	0
**ROME POLICLINICO GEMELLI**	0	0	2
**BARI**	0	2	0
**PADUA**	1	0	0
**TOT**	39	25	20

**Figure 1:**
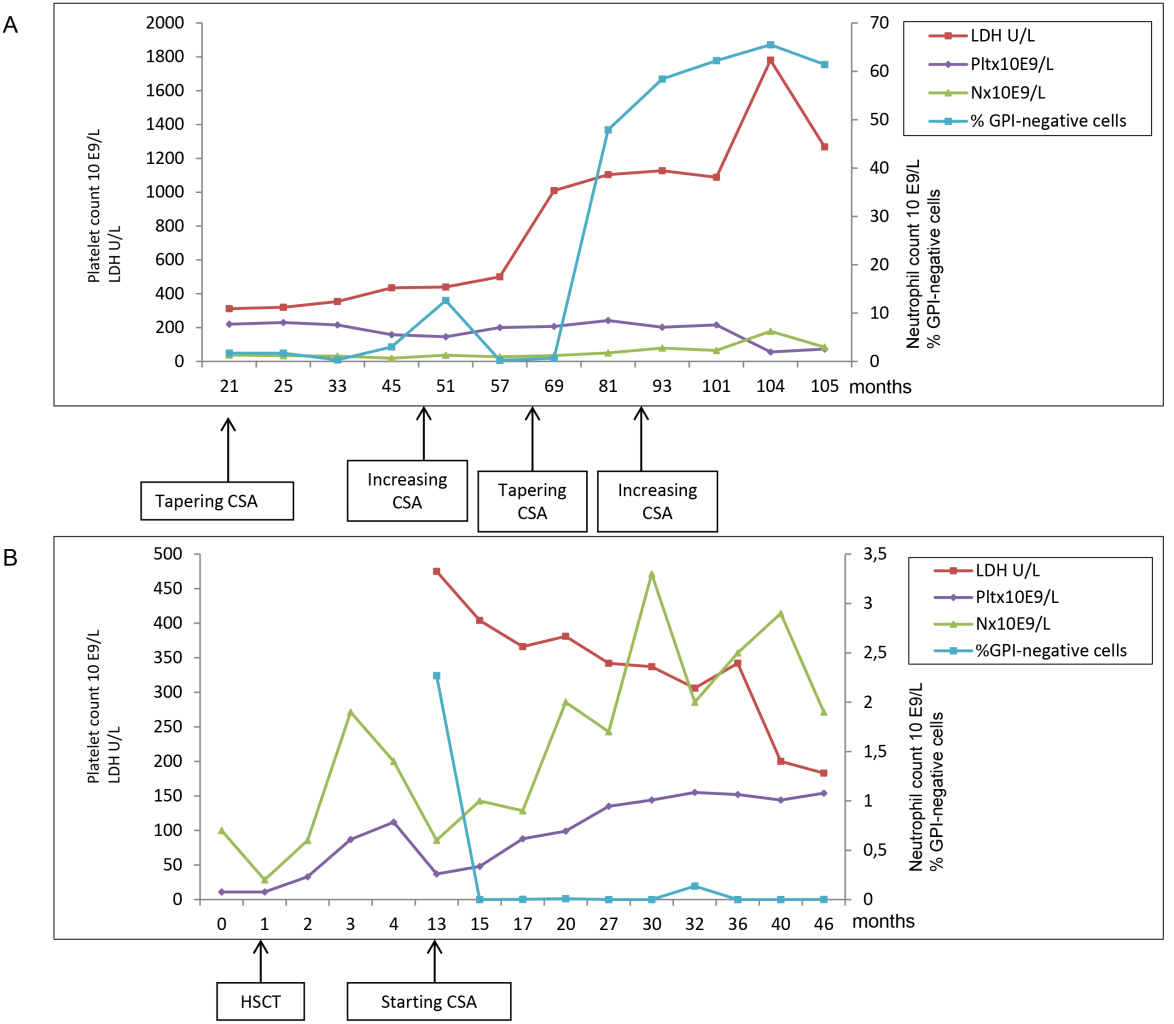
Follow up of GPI-negative populations in two representative aplastic anemia (AA) patients. A: AA patient in IST. Variations of GPI-negative population size. The patient developed a frank PNH without hemoglobinuria. B: AA patient treated at first line with related HSCT and entered in the study at time of AA relapse after graft failure (falling of peripheral blood counts and autologous reconstutition with 100% recipient chimerism, after a previously full donor graft) A GPI-negative population was present at AA relapse and disappeared after starting immune-suppressive therapy with cyclosporine (persistent autologous reconstutition 100% recipient chimerism; persistent complete remission obtained).

Eighty-four AA patients described in the multicentre study. Thirty-nine were studied from diagnosis, 25 during IST, 20 off therapy. One more selected case (from Monza, not shown in this table) was analysed at relapse after HSC transplantation (HSCT) and is shown in Fig. 1B.

*The follow-up of one out of these five patients is shown in Fig. 1A.

Among 30 patients from one single centre, 6 were newly enrolled, whereas for the other 24 (enrolled before 2008) we present an update of previously published data [13]. Thirty-nine were studied from diagnosis, 25 during IST, 20 off therapy and one selected case was analyzed for the first time at relapse after first line HSC transplantation (HSCT). Among the patients followed since diagnosis, 8 received an HLA matched sibling donor HSCT as first line therapy, one patient had spontaneous remission without therapy, 28 patients were treated with IST according to EBMT protocols (cyclosporine A (CSA), anti-thymocyte globulin ± granulocyte colony stimulating factor). Horse anti-thymocyte globulin (hATG) was utilized in 14 patients, rabbit ATG (rATG) was utilized in 14 patients (all diagnosed after 2008, when hATG was not available in Italy). Two patients with NSAA were treated with CSA alone.

Samples in EDTA were centralized in the Turin laboratory and analyzed within 24 hours. Peripheral blood GPI-negative cells were detected by lack of CD59 expression on granulocytes by two-colour FCA for CD59 (clone p282-FITC Becton-Dickinson) and CD11b (clone D12-PE Becton-Dickinson); 10^5^ cells were analysed, for a total of 1104 tests. The presence of a CD11b+/CD59− population >0.15% was defined as abnormal; the cut off value was established by evaluating 87 normal controls (GPI-negative population: median = 0.001%, mean+3SD = 0.14%) (13). The same flow cytometry technique was utilized in all the analysis to obtain comparable data, however since 2009, two-colour FCA results were confirmed by more sensitive techniques with three or six-colour sequential gating analysis for CD45/33/66b or CD45/33/15/24/14/FLAER.

## Results

A GPI-negative population was observed in 16 out of 39 (41%) patients at diagnosis (population size 0.17–10.4%; absolute count 0.05–437.2 GPI-negative cells/µl), in 12 out of 25 patients (48%) studied during IST (population size 0.16–65.5%; 1.4–1735 GPI-negative cells/µl) and in 9 out of 20 patients (45%) studied when off-therapy (population size 0.16–4.0%; 8.5–155 GPI-negative cells/µl).

In 33 patients (16 followed since diagnosis, 9 in IST, 8 off-therapy), the GPI-negative population was sporadic or intermittent, whereas in 13 patients (9 followed since diagnosis, 3 in IST, 1 off-therapy) it persisted for more than 3 sequential controls. The GPI-negative population size was significantly smaller in the persistent group than in the sporadic (median 0, 31%, range 0.16–3.0% versus 0.74%, range 0.16–6.0%, p<0.05). In 8 out of the 23 GPI-positive patients at diagnosis, a GPI-negative population (size 0.16–1.7%; 0.1–26.4 GPI-negative cells/µl) appeared later during IST.

Table 2 summarizes the evaluation of GPI-negative populations, neutrophil counts and the response to therapy in the 30 patients followed since diagnosis and treated with IST.

**Table 2:** Neutrophils, GPI-negative population and outcome of 30 AA patients followed since diagnosis and treated with IST as first line therapy.

		T0, pre-IST			T180, post IST			
PATIENTS ATDIAGNOSIS	First line treatment	Nx10 E9/L	% GPI-negative cells	GPI-negative cells/µL	Nx10 E9/L	% GPI-negative cells	GPI-negative cells/µL	Response to therapy
**D1**	**hATG+CSA**	0.40	0.1700	0.68	2.10	0.1900	3.99	NR
**D2**	**hATG+CSA**	0.30	0.4000	1.20	0.58	0.0000	0.00	NR
**D3**	**hATG+CSA**	0.60	0.3000	1.80	1.40	0.2000	2.80	NR
**D4**	**hATG+CSA**	0.07	0.7364	0.51	2.11	0.0050	0.10	PR
**D5**	**hATG+CSA**	1.00	0.3000	3.00	1.43	1.1000	15.73	PR
**D6**	**hATG+CSA**	0.01	0.5000	0.05	4.64	0.0000	0.00	PR
**D7**	**hATG+CSA**	0.40	0.0000	0.00	1.90	0.0000	0.00	NR
**D8**	**hATG+CSA**	1.10	0.0000	0.00	5.90	0.2000	11.80	NR
**D9**	**hATG+CSA**	0.05	0.1000	0.05	1.48	0.1600	2.36	PR
**D10**	**hATG+CSA**	0.50	0.0600	0.30	2.04	0.3120	6.36	PR
**D11**	**rATG+CSA**	0.67	2.2000	14.74	1.05	0.2550	2.67	NR
**D12**	**rATG+CSA**	0.80	0.4800	3.84	1.30	0.2800	3.64	NR
**D13**	**rATG+CSA**	0.36	0.0055	0.01	0.06	0.1840	0.11	NR
**D14**	**rATG+CSA**	0.35	0.0000	0.00	0.42	0.4984	2.09	NR
**D15**	**rATG+CSA**	0.98	0.0070	0.06	1.00	0.2000	2.00	PR
**D16**	**rATG+CSA**	0.40	0.0370	0.14	3.83	0.1562	5.98	CR
**D17**	**hATG+CSA**	0.80	0.5500	4.40	0.60	0.0000	0.00	CR
**D18**	**hATG+CSA**	0.40	0.0000	0.00	0.40	0.0700	0.28	PR
**D19**	**hATG+CSA**	0.40	0.0000	0.00	2.80	0.0060	0.16	CR
**D20**	**rATG+CSA**	0.40	0.0850	0.34	0.06	0.1100	0.06	NR
**D21**	**rATG+CSA**	0.10	0.0020	0.02	2.30	0.7985	18.36	NR
**D22**	**rATG+CSA**	0.40	0.0269	0.10	0.40	0.0000	0.00	NR
**D23**	**CSA**	0.40	0.0328	0.13	0.60	0.0000	0.00	NR
**D24**	**CSA**	0.10	0.0000	0.00	0.80	0.0000	0.00	CR
**D25**	**rATG+CSA**	1.70	2.7640	46.98	0.60	1.1457	6.87	PR
**D26**	**rATG+CSA**	2.20	0.0000	0.00	2.50	0.0000	0.00	NR
**D27**	**rATG+CSA**	0.40	0.0450	0.18	0.40	0.0000	0.00	NR
**D28**	**rATG+CSA**	0.01	0.6500	0.06	0.01	0.6600	0.06	NR
**D29**	**rATG+CSA**	0.08	0.0000	0.00	2.27	0.0000	0.00	NR
**D30**	**hATG+CSA**	0.50	0.0326	0.16	1.40	0.0000	0.00	PR

hATG = horse ATG; rATG = rabbit ATG; CSA = cyclosporin. In patients treated with hATG evaluation at 180 days showed 2 complete responders (CR, 14%), 7 partial-responders (PR, 50%) and 5 non-responders (NR, 36%), whereas in patients treated with rATG evaluation at 180 days showed 1 CR (7%), 2 PR (14%), 11 NR (79%) (2-tailed Fisher test p = 0.0542).

Among the 11 patients GPI negative at diagnosis, CR and PR were respectively 1 (9%) and 4 (36%), whereas NR were 6 (55%). Among the 19 patients GPI positive at diagnosis CR and PR were respectively 3 (16%) and 5 (26%), whereas NR were 11 (58%) (2-tailed Fisher test p = 1). In all the 4 GPI-negative patients who were treated with rATG the clone persisted at day +180, whereas it disappeared in 4/7 patients receiving hATG up-front.

We did not observe a significant difference in the response to IST between patients GPI-negative and GPI-positive at diagnosis (2-tailed Fisher test = 1). No significant difference in the response to IST was also observed between the patients GPI-positive and the subgroup of patients with persistent GPI-negative populations. (2-tailed Fisher test = 1) The response rate to IST did not differ between the 8 patients GPI-positive at diagnosis who later developed GPI-negative populations (1 CR, 3PR) and the 11 who remained GPI-positive (2 CR, 2 PR). (2-tailed Fisher test p = 0.65). The decrease or disappearance of GPI-negative cells did not simply reflect the recovery of neutrophil counts, since there was not a significant correlation between clone size and neutrophil counts at diagnosis and at time +180. (Δ neutrophils versus Δ% of GPI-negative population: R = 0.15; Δ neutrophils versus Δ absolute count of GPI-negative population: R = 0.44). The response rate to IST did not differ also between the 7 patients GPI-negative at diagnosis who remained GPI-negative at day +180 (2 PR) and the 4 patients GPI-negative at diagnosis in whom the GPI-negative population disappeared at day +180 (1 CR, 2 PR) (2-tailed Fisher test p = 0.24). In patients GPI-negative at diagnosis who remained GPI-negative at day +180 we observed a trend towards a reduction of the clone size.

Among the 25 patients studied during IST, two had a GPI-negative population that appeared during the tapering of CSA. One of these patients is described in Figure 1A. The other, a GPI-positive patient from the Monza Centre (see Table 1) had a GPI-negative population of 2.8% that appeared during CSA tapering.

In 2 patients previously GPI-positive, a GPI-negative population appeared at time of relapse when off therapy (update of previously published data). In the selected patient treated with first line HSCT, a GPI-negative population was present after graft failure (autologous reconstitution 100% recipient) at the time of AA relapse and disappeared after starting IST with CSA (Figure 1B).

Excluding the 2 patients with a GPI-negative population >10%, we did not observe a significant correlation between LDH levels and GPI-negative population size (R = 0.019) In the 2 patients (patients AAa and AAb) with a GPI-negative population >10%, mild to moderate hemolysis was observed. Patient AAa who developed a frank PNH without hemoglobinuria is described in details in Figure 1A. Patient AAb showed at diagnosis: Hb = 73 g/L, reticulocytes = 123.0×10^9^/L, LDH = 2448 U/L, without hemoglobinuria. He was treated with HSCT from a sibling matched donor as first-line therapy, with disappearance of the GPI-negative clone. He has been considered as AA/PNH. No thrombotic event was reported in the whole cohort of AA patients.

## Discussion

In the present study conducted on a large number of pediatric AA patients we observed a significant rate of minor GPI-negative populations at diagnosis similar to observations in adults [2], [9], [10], [12]. Noteworthy, 2 patients (one at diagnosis and one during follow up) showed major (>10%) GPI-negative populations with moderate hemolysis and without hemoglobinuria, and were considered as AA/PNH.

In agreement with other reports [11], [12], [14] and with our previous study [13], we did not find a positive correlation between the presence of pre-treatment GPI-negative populations and favourable response to IST. The appearance of a GPI-negative population in patients previously GPI-positive at diagnosis, reported as uncommon by others [15], was seen in a high proportion (42%) of our IST-treated patients. The observation of fluctuation of GPI-negative populations during IST or the appearance of GPI-negative populations in previously GPI-positive patients during IST or even off therapy, suggests complex interactions between stem cell immune-mediated damage and immune-suppression, such as transient or persistent reactivation of the immune attack. Moreover, without a mutation analysis is impossible to state if the GPI-negative cells observed in the periodical analysis of the same patient belong to the same or different clones.

The patients with a persistent GPI-negative population are an interesting subgroup that might be biologically different from the “sporadic”. Unexpectedly, the size of GPI-negative population was smaller in the “persistent” compared to the “sporadic” patients, while there was no difference in the response to IST.

Although the number of patients receiving IST upfront does not enable definitive conclusions, we observed that in hATG-treated subjects GPI-negative populations disappeared more frequently than in those who were given rATG. Occasionally, the appearance of a GPI-negative populations was observed during CSA tapering or at AA relapse. One such example is the patient shown in Figure 1A who before developing a frank hemoglobinuria during follow up, showed an increased GPI-negative population at the first CSA tapering. This GPI-negative population disappeared for 12 months after the CSA dose was increased. Published data about changes in GPI-negative populations size during IST are limited and this finding may be important clinically. Thrombosis is the most severe complication of PNH. We did not observe thrombosis in the two AA/PNH patients neither in the patients with minor GPI-negative populations.

As PNH is extremely rare in children, a considerable delay in diagnosis has been previously described in literature [16], [17], supporting the utility of a careful monitoring with flow cytometry of patients at risk for PNH, as AA patients [4], [18]. In our study, positive laboratory signs of hemolysis were observed in patients who had GPI-negative neutrophils greater than 10%, whereas LDH levels were not informative about the presence of minor GPI-negative populations. Therefore, our experience suggests that a careful monitoring for laboratory signs of hemolysis may be sufficient for the diagnosis of symptomatic PNH in AA patients, whereas a systematic flow cytometry follow up represents a more complex and expensive approach. However, the possibility, even if very rare, of a frank PNH without signs of hemolysis has been reported. [19], [20] and in severely aplastic patients laboratory markers of hemolysis might not be altered in presence of PNH clones large as percentage but small as absolute number.

Finally, the biological role of minor GPI-negative populations in AA and their relationship with immune-suppression may need further investigation.
